# The open diffusion data derivatives, brain data upcycling via integrated publishing of derivatives and reproducible open cloud services

**DOI:** 10.1038/s41597-019-0073-y

**Published:** 2019-05-23

**Authors:** Paolo Avesani, Brent McPherson, Soichi Hayashi, Cesar F. Caiafa, Robert Henschel, Eleftherios Garyfallidis, Lindsey Kitchell, Daniel Bullock, Andrew Patterson, Emanuele Olivetti, Olaf Sporns, Andrew J. Saykin, Lei Wang, Ivo Dinov, David Hancock, Bradley Caron, Yiming Qian, Franco Pestilli

**Affiliations:** 1 Neuroinformatics Laboratory, Center for Information Technology, Fondazione Bruno Kessler, via Sommarive 18, 38123 Trento, Italy; 2 Center for Mind/Brain Sciences (CIMeC), University of Trento, via Delle Regole 101, 38123 Trento, Italy; 3 Pestilli Lab. Department of Psychological and Brain Sciences, Program in Cognitive Science, Indiana University Bloomington, 1101 E 10th Street, Bloomington, Indiana 47405 USA; 4 Pestilli Lab. Department of Psychological and Brain Sciences, Indiana University Bloomington, 1101 E 10th Street, Bloomington, Indiana 47405 USA; 5 Instituto Argentino de Radioastronomía (CCT-La Plata, CONICET; CICPBA), CC5 V, Elisa, 1894 Argentina; 6 Facultad de Ingeniería, Universidad de Buenos Aires, Buenos Aires, C1063ACV Argentina; 7 Pestilli Lab. Department of Psychological and Brain Sciences, Program in Neuroscience, Indiana University Bloomington, 1101 E 10th Street, Bloomington, Indiana 47405 USA; 8 Department of Psychological and Brain Sciences, Programs in Neuroscience and Cognitive Science, and Indiana Network Science Institute, Indiana University Bloomington, 1101 E 10th Street, Bloomington, Indiana 47405 USA; 9 Department of Intelligent Systems Engineering, Programs in Neuroscience and Cognitive Science, Indiana University Bloomington, 700N Woodlawn Ave, Bloomington, Indiana 47408 USA; 10 Indiana University School of Medicine, Departments of Radiology and Imaging Sciences and Medical and Molecular Genetics, and the Indiana Alzheimer Disease Center, Indiana University, 355 W 16th St., Indianapolis, Indiana 46202 USA; 11 Department of Psychological and Brain Sciences and Pervasive Technology Institute, University Information Technology Services, Indiana University, 1101 E 10th Street, Bloomington, IN 47405 USA; 12 Pervasive Technology Institute, Indiana University Bloomington, 2709 E 10th Street, Bloomington, IN 47408 USA; 13 Departments of Psychiatry and Behavioral Sciences and Radiology, Northwestern University Feinberg School of Medicine, 710N. Lake Shore Drive, Abbott Hall 1322, Chicago, IL 60611 USA; 14 Statistics Online Computational Resource (SOCR), Center for Complexity of Self-Management in Chronic Disease (CSCD), Health Behavior and Biological Sciences, Michigan Institute for Data Science (MIDAS), University of Michigan, Ann Arbor, MI 49109 USA; 15 Pestilli Lab. Indiana University School of Optometry and Program in Neuroscience, Indiana University Bloomington, 1101 E 10th Street, Bloomington, Indiana USA; 16 Pestilli Lab. Department of Psychological and Brain Sciences, Engineering, Computer Science, Programs in Neuroscience and Cognitive Science, School of Optometry, and Indiana Network Science Institute, Indiana University Bloomington, 1101 E 10th Street, Bloomington, Indiana 47405 USA

**Keywords:** Network models, Brain imaging, Computational science, Cognitive neuroscience, Magnetic resonance imaging

## Abstract

We describe the Open Diffusion Data Derivatives (O3D) repository: an integrated collection of preserved brain data derivatives and processing pipelines, published together using a single digital-object-identifier. The data derivatives were generated using modern diffusion-weighted magnetic resonance imaging data (dMRI) with diverse properties of resolution and signal-to-noise ratio. In addition to the data, we publish all processing pipelines (also referred to as open cloud services). The pipelines utilize modern methods for neuroimaging data processing (diffusion-signal modelling, fiber tracking, tractography evaluation, white matter segmentation, and structural connectome construction). The O3D open services can allow cognitive and clinical neuroscientists to run the connectome mapping algorithms on new, user-uploaded, data. Open source code implementing all O3D services is also provided to allow computational and computer scientists to reuse and extend the processing methods. Publishing both data-derivatives and integrated processing pipeline promotes practices for scientific reproducibility and data upcycling by providing open access to the research assets for utilization by multiple scientific communities.

## Abstract (summary)

**Table:** 

Design Type(s)	data integration objective • database creation objective
Measurement Type(s)	brain
Technology Type(s)	magnetic resonance imaging
Factor Type(s)	Voxel Size
Sample Characteristic(s)	Homo sapiens • brain

Machine-accessible metadata file describing the reported data (ISA-Tab format)

## Background & Summary

In the past decade, efforts in large-scale neuroimaging data collection have redirected research attention towards effective practices for data sharing, reuse, standardization and secondary data analyses. Some of the most notable examples include projects such as the Human Connectome Project^1–3^, UK Biobank^4,5^, ADNI^6^, INDI^7^, ABCD^8^, CamCAN^9^ and OpenfMRI (recently rebranded as OpenNeuro)^10^. These projects have served as the bellwether for data-sharing in a growing culture focused on advancing methods for open big-data reproducible science^11–15^. Similar efforts for large-scale shared collections can, in principle, promote the establishment of best practices for measurements standards, and neuroinformatics methods, thereby contributing to a new generation of Big Data neuroscience research^16,17^.

The use of the same data can be different across scientific communities. Data sharing can increase data value by promoting reuse for purposes beyond those of the original project; a process we call *data upcycling*^18^. As part of this upcycling process, data derivatives (secondary products generated by the various data analyses processes) can become useful data for scientific communities outside of the community of origin. Succinctly stated, various scientific communities may have different interests in reusing brain data. For example, a white matter segmentation can be used by computer scientists for methods development^19–26^ or by neuroscientists to understand the brain^27–32^. Indeed, data can be reused for several applications not foreseen in the original study^33^, for example, to develop theoretical frameworks^34^, new algorithms^22,35,36^, advance data visualization practices^37–39^, and even for statistical validation of results^40–42^. The process of upcycling can help to extract additional value from openly available data sets, thereby returning continuing dividends from the initial resource investments.

We propose a unique approach to brain data upcycling by presenting the Open Diffusion Data Derivatives (O3D), a repository composed of both data-derivatives and their associated processing pipelines, bundled together and referenced by a single digital object identifier (DOI^43^). The O3D data were derived from anatomical (T1-weighted), diffusion-weighted magnetic resonance imaging (dMRI) data and tractography methods. The O3D data were obtained from previously published high-quality, high-resolution dMRI data^1,40,44,45^ and processing pipelines^22,40,45–47^. The dataset is comprised of (1) the minimally preprocessed dMRI data files (12 brains from three different datasets with different properties of signal-to-noise ratio and resolution) and (2) a large set of diverse data derivatives comprising 360 tractograms, 7,200 segmented major tracts, and 720 connection matrices. The total size of the O3D repository is approximately 1.79 Terabytes of data derivatives.

Diffusion-weighted magnetic resonance imaging (dMRI) and tractography allow measuring structural connectomes, white matter macro-anatomy, and microscopic tissue properties from the living human brain. These techniques have revolutionized our understanding of how brain networks and the brain’s white matter impact human behavior, in health and disease^29,30,48–58^. Neurotractography techniques provide fundamental insights about the human brain, and yet there is much work that remains to be done to map the human connectome^40,59–63^. Because of the complexity of these methods, the success of the modern scientific enterprise in mapping the human connectome almost certainly depends on transdisciplinary contributions from multiple communities – from Psychology and Neuroscience to Mathematics and Statistics, as well as to Computer Science and Engineering. For this reason open scientific discovery and collaborative sharing of methods, software, and data are of paramount importance^16,64,65^.

In addition to data, the processing pipelines used to generate the O3D data are made available on the brainlife.io platform as a series of “open services,” hereafter referred to as brainlife.io Apps or simply Apps. We define open services as self-contained processing applications embedded and reusable in a cloud platform environment. The brainlife.io platform allows running said Apps to process data available within the platform itself^66–68^. The concept of open service is akin to that of the Brain Imaging Data Structure Applications^69^ as also introduced previously by others^70^. The brainlife.io Apps used below follow a generalized and light-weight specification as to allow usage with diverse combinations of software from multiple libraries, such as FSL^71^, FreeSurfer^72^, DIPY^73^, Nipype^74^, LiFE^22,40^, AFQ^75^, MRtrix^76^, and AFNI^77^. These Apps can be containerized and made reproducible using technologies such as Docker^78^ and Singularity^79^. Alternatively, brainlife.io Apps can also run without containerization on software environments compatible with NeuroDebian^80^. The brainlife.io platform currently utilizes a mixture of public (jetstream-cloud.org^81,82^; opensciencegrid.org^68^), commercial (azure.microsoft.com and cloud.google.com), as well as institutional (carbonate.uits.iu.edu) computing resources. The platform is a registered DataCite center (search.datacite.org/data-centers/brainl.iu), member of the fairsharing.org catalogue (see^83^), as well as registered project on both the NeuroImaging Tools and Resources Collaboratory (http://www.nitrc.org/projects/brainlife_io) and scicrunch.org (RRID: SCR_016513).

Publication records on brainlife.io/pubs, such as O3D, are preserved for at least ten years since latest use, and comply with the schema.org metadata specification to promote maximum discoverability and respect of the FAIR principles^84^. The complete list of brainlife.io Apps used to generate the O3D data are preserved as part of the repository^43^. These Apps are both provided as preserved files to allow accessing of the code version used to generate the specific O3D data, and can be reused for future research. The brainlife.io publication and preservation strategy is resilient to version changes likely to occur over time for each App or dataset. A full description of the brainlife.io platform and Apps is beyond the scope of this data descriptor; more information can be found here: brainlife.io/docs.

Using the O3D project, investigators can either process new data using the same pipelines used to generate the core O3D repository or they can download data derivatives processed at different stages along the series of steps taken to generate tractography, white matter tracts, and connectivity matrices. Additionally, new data can in principle be uploaded using the brainlife.io web-portal and used to generate new results. Data can also be downloaded using a simple web or command line interfaces format as BIDS (Brain Imaging Data Structure)^85,86^. Finally, open source code and containers implementing the processing pipelines can be found at github.com/brainlife and hub.docker.com/u/brainlife.

The O3D repository is unique in that it focuses on publishing repeated-measures data-derivatives for tractography, white matter tracts, and structural connectome matrices–all associated with open services publishing reproducible data processing pipelines and workflows. The O3D dataset provides a means for computational test-retest quantification^41,87–89^ and reproducibility. To generate the data derivatives, three tractography algorithms were used ten times on the same data source (individual brain). Due to stochasticity of such algorithms, the results for each of these are slightly different. The number of repeats has been previously shown to allow measuring variability and reliability of connectome mapping methods^21,22,40^. The tractography results were evaluated using state-of-the-art methods^22,40^ and compared against classical neuroanatomy atlases used to segment the major human white matter tracts^75,90^. Finally, a series of connection matrices (i.e. brain networks) were generated using standard cortical parcellation methods^91^. Three example scenarios can be used to demonstrate transdisciplinary applications and show how investigators from different communities can utilize the O3D core set. First, investigators developing network science algorithms^35,63,92–94^ might have an interest in demonstrating the applicability or efficacy of their methods on brain network data, but lack skills to process the raw diffusion data into connectivity matrices. The data derivatives provide an easily accessible point of entry by making available unthresholded brain connection matrices built using data from multiple individuals and different tracking methods. Second, investigators studying white matter neuroanatomy, or developing software for automated segmentation of white matter tracts, can use the data derivatives as complex test objects to compare the results of new algorithms with the state of the art reference set represented by O3D^25,95–97^. Finally, the data derivatives can be an essential education and training resource. It may be used by students and trainees in the neural and clinical sciences to learn about neuroanatomy or to develop practical analytic skills. All O3D data is compatible with most major neuroimaging software packages and can be conveniently loaded, processed and visualized^40,71–73,75,76^.

The present descriptor introduces the O3D repository and some of the brainlife.io publication mechanisms, as necessary to describe the repository. The O3D reference repository will allow investigators from multiple scientific communities to explore brain data, perform visualization experiments, and replicate the data derivatives without having to first learn a full processing pipeline. This lowers the barrier of entry to computational neuroimaging, with the potential to advance algorithmic development, increase the involvement of underrepresented scholars, and to facilitate training and validation^16,98^. The repeated measure data derivatives we plan to distribute as part of O3D will appeal to a diverse range of research interests because of the extensive know-how necessary to generate them. Consequently, they can be used by communities of basic, clinical, translational and computational scientists including neuroscientists, students and trainees early in their careers^16,98,99^.

## Methods

### Data sources

Three diffusion-weighted Magnetic Resonance Imaging datasets (dMRI) were used to generate all the derivatives in the initial repository layout, from publicly available sources (https://purl.stanford.edu/rt034xr8593^45^, https://purl.stanford.edu/ng782rw8378^40,45^, https://purl.stanford.edu/bb060nk0241^27^ and https://www.humanconnectome.org/data^2,100^).

#### Stanford dataset (STN)

We used data collected in four subjects at the Stanford Center for Cognitive and Neurobiological Imaging with a 3T General Electric Discovery 750 MRI (General Electric Healthcare), using a 32-channel head coil (Nova Medical). dMRI data had whole-brain coverage and were acquired with a dual-spin echo diffusion-weighted sequence, using 96 diffusion-weighting directions and gradient strength of 2,000 s/*mm*^2^ (TE = 96.8 ms). Data spatial resolution was set at 1.5 mm isotropic. Each dMRI is the average of two measurements (NEX = 2). Ten non-diffusion-weighted images (b = 0) were acquired at the beginning of each scan^40,45,47^.

#### Human connectome project datasets (HCP3T and HCP7T)

We used data collected in 8 subjects from the Human Connectome Project, using Siemens 3T and 7T MRI scanners. Only measurements from the 2,000 s/*mm*^2^ shell were extracted from these data and used to generate the data derivatives in our repositories. Data from the 3T and 7T scanners have different properties of resolution (e.g., HCP3T, 90 gradient directions, 1.25 *mm* isotropic resolution and HCP7T, 60 gradient directions, 1.05 *mm* isotropic resolution) and have been described before along with the processing methods used for data preprocessing^44,100–102^.

### Data preprocessing

We developed a series of steps to process the anatomical and dMRI data files in a standardized manner for publication as part of the O3D repository. All original data were oriented to the plane defined by the Anterior and Posterior Commissure and the 2,000 s/mm^2^ shell was selected and utilized for the subsequent analyses. All MRI data were oriented in Neurological coordinates (Left-Anterior-Superior) and the bvecs files were oriented accordingly. The brainlife.io Apps implementing these operations can be found at^103–105^ (see also Tables 1 and 2). No additional denoising, eddy current or head movement correction was applied beyond that performed by the data originators.

**Table 1:** List of the current Apps implementing the processing steps used to generate O3D to be re-used on brainlife.io as open services.

App goal	DOIs of each O3D App as service on brainlife.io
1. ACPC alignment of T1	https://doi.org/10.25663/bl.app.16^103^
2. Split dMRI shells	https://doi.org/10.25663/bl.app.17^104^
3. dMRI data preprocessing	https://doi.org/10.25663/bl.app.3^105^
4. Brain parcellation	https://doi.org/10.25663/bl.app.0^112^
5. Tractography	https://doi.org/10.25663/bl.app.59^113^
6. Tractography evaluation	https://doi.org/10.25663/bl.app.1^115^
7. Network neuroscience	https://doi.org/10.25663/bl.app.47^121^
8. White matter classification (WMC)	https://doi.org/10.25663/bl.app.13^116^
9. Refine white matter classification	https://doi.org/10.25663/bl.app.11^117^
10. WMC file format conversion	https://doi.org/10.25663/brainlife.app.127^118^
11. Tractogram file format conversion	https://doi.org/10.25663/brainlife.app.132^139^

**Table 2:** List of software repositories with the code version of the scripts implementing the O3D Apps.

App goal	URLs of each O3D App code repository on GitHub.com
1. ACPC alignment of T1	https://github.com/brainlife/app-acpcART
2. Split dMRI shells	https://github.com/brainlife/app-splitshells
3. dMRI data preprocessing	https://github.com/brainlife/app-dtiinit
4. Brain parcellation	https://github.com/brainlife/app-freesurfer
5. Tractography	https://github.com/brainlife/app-tracking
6. Tractography evaluation	https://github.com/brainlife/app-life
7. Network neuroscience	https://github.com/brainlife/app-networkneuro
8. White matter classification (WMC)	https://github.com/brainlife/app-tractclassification
9. Refine white matter classification	https://github.com/brainlife/app-AFQclean
10. WMC file format conversion	https://github.com/brainlife/app-wmctotrk
11. Tractogram file format conversion	https://github.com/brainlife/app-convert-tck-to-trk

### Voxel signal reconstruction and tractography

White matter fascicles tracking was performed using MRtrix 0.2.12^76^. White- and gray-matter tissues were segmented with Freesurfer^72^ using the T1-weighted MRI images associated to each individual brain, and then resampled at the resolution of the dMRI data. Only voxels identified primarily as white-matter tissue were used to constrain tracking. We used three different tracking methods: (A) tensor-based deterministic tracking^106,107^, (B) Constrained Spherical Deconvolution (CSD) -based deterministic tracking^76,108^, and (C) CSD-based probabilistic tracking^108,109^. Maximum harmonic orders *L*_max_ = 10 (STN, HCP3T) and *L*_max_ = 8 (HCP7T) were used^110,111^. Other parameters settings used to perform tracking were: step size: 0.2 mm; maximum length, 200 mm; minimum length, 10 mm. The fiber orientation distribution function (*f*_ODF_) amplitude cutoff, was set to 0.1, and for the minimum radius of curvature we adopted the default values, fixed by MRtrix for each kind of tracking: 2 mm (DTI deterministic), 0 mm (CSD deterministic), 1 mm (CSD probabilistic). We generated repeated measures of tractography derivatives by computing 10 candidate whole-brain fascicles groups for each individual brain using 500,000 fascicles each. Apps implementing the methods can be found at^112–114^.

### Tractography evaluation

We used the Linear Fascicle Evaluation method (LiFE)^40^ to optimize whole-brain tractograms implemented using the recently proposed ENCODE model^22^. The LiFE method identifies fascicles that successfully contribute to prediction of the measured dMRI signal. It has been shown that only a percentage of the total number of fascicles generated through a single tractography method is supported by the properties of given dataset^40,47^. Because of this we removed all fascicles making no significant contribution to explaining the diffusion measurements. The percentage of streamlines retained in these optimized fascicles groups ranged between 10–20% (STN), 15–35% (HCP3T) and 20–40% (HCP7T). Apps implementing the method can be found at^115^.

### White matter tracts segmentation

Twenty major human white matter tracts were segmented using the Automating Fiber-tract Quantification (AFQ) method^75^. An additional step refined the segmented tracts by removing the fiber outliers. The following tracts were segmented: left and right Anterior Thalamic Radiation (ATRl and ATRr), left and right corticospinal tract (CSTl and CSTr), left and right Cingulum - Cingulate gyrus (CCgl and CCgr), left and right Cingulum - Hippocampus portion (CHil and CHir), left and right Inferior Fronto-Occipital Fasciculus (IFOFl and IFOFr), left and right Inferior Longitudinal Fasciculus (ILFl and ILFr), left and right Superior Longitudinal Fasciculus (SLFl and SLFr), left and right Uncinate Fasciculus (UFl and UFr), left and right Superior Longitudinal Fasciculus - Temporal part (often referred to as the “arcuate fasciculus”, SLFTl and SLFTr), Forceps Major (FMJ), and Forceps Minor (FMI). Each tract was stored in trackvis file format. Apps implementing the method can be found at^116–118^.

### Connection matrix construction

We used tractograms evaluated by the LiFE method to build connectivity matrices. Connectivity matrices were built for each fascicle groups using the 68 cortical regions from the Desikan Killiany atlas, segmented in each individual using T1w MRI images and FreeSurfer^72,91^. Fascicles terminations were mapped onto each of the 68 regions. All fibers connecting pairs of brain regions were identified and collected. Adjacency matrices were built using two measures: (A) *count*^119^, by computing the number of fascicles connecting each unique pair of regions, (B) *density*, by computing the density of fibers connecting each unique pair – computed as twice the number of fascicles between regions divided by sum of the number of voxels in the two atlas regions^88,94,119,120^. Apps implementing the method can be found at^121^.

### Open service for reproducible neuroscience: brainlife.io/apps

We provide the full set of scripts used to process the O3D repository, both as open services, also referred to as Apps, that can be run on the brainlife.io platform (Table 1), as well as, code, scripts used to implement each App available on github.com/brainlife (Table 2). Whereas the code can be downloaded for running locally the scripts, the Apps are embedded in the brainlife.io platform and can be reused to directly process data avoiding the needs of installing software.

Brainlife.io Apps can be improved over time by users or developers and for this reason their implementation can change. As such, brainlife.io uses github.com to keep track of App versions. We note that whereas the DOIs for the Apps reported in Table 1 direct users to the most recent version of each App available on the platform, the URLs in Table 2 direct users to the specific version of the code used for the preprocessing used to generate the published O3D dataset. To fully support the reproducibility of the O3D publication we preserve for each release both the data and a snapshot of the code for each App. The O3D Apps preserved with the original code version used to generate the repository is reported in^43^.

## Data Records

Preserved O3D data and Apps can be downloaded at the web URL reported in^43^. Upon download, data will automatically be organized as brainlife.io DataTypes (brainlife.io/docs/user/datatypes and brainlife.io/datatypes) as well as according to the specification defined by the Brain Imaging Data Structure (BIDS)^85^. We note that, currently, BIDS does not officially provide a complete specification for diffusion-weighted magnetic resonance imaging and tractography derivatives.

According to the provisional BIDS specification for data derivatives (https://goo.gl/aFJ6vS), we have organized the files within folders, where each folder name refers to the name of the brainlifle.io App used to generate the files. The file naming convention adopted for the folders is based on three tokens: (A) The name of the github.com organization (e.g., brainlife); (B) the name of the repository of the App (e.g., app-life). All files generated by an App are aggregated in subfolders, one for each subject. Following the BIDS convention: (1) each file name includes a descriptor (_desc-) referring a unique brainlife.io identifier, (2) additional information on the brainlife.io DataType reported in filename by tags (_tag-), (3) the repeated measures are denoted by the keyword run (_run-), (4) the last token of the file names indicates the BIDS datatype (e.g., _dwi-), (5) the suffix denotes the file format (e.g., .nii.gz), and (6) metadata are recorded as a JSON file^122^.

### Source data

The source files of anatomy uploaded to brainlife.io are stored as follow:

upload/sub-{}/anat/

sub-{}_tag-acpcaligned_desc-{}_T1w.json

sub-{}_tag-acpcaligned_desc-{}_T1w.nii.gz

The source files of diffusion MRI uploaded from Stanford to brainlife.io are stored as follow:

upload/sub-{}/dwi/

sub-{}_tag-normalized_tag-singleshell_desc-{}_dwi.json

sub-{}_tag-normalized_tag-singleshell_desc-{}_dwi.nii.gz

sub-{}_tag-normalized_tag-singleshell_desc-{}_dwi.bvals

sub-{}_tag-normalized_tag-singleshell_desc-{}_dwi.bvecs

The source files of diffusion MRI uploaded from HCP to brainlife.io are stored as follow

brain-life.app-splitshells/sub-{}/dwi/

sub-{}_tag-normalized_tag-singleshell_desc-{}_dwi.json

sub-{}_tag-normalized_tag-singleshell_desc-{}_dwi.nii.gz

sub-{}_tag-normalized_tag-singleshell_desc-{}_dwi.bvals

sub-{}_tag-normalized_tag-singleshell_desc-{}_dwi.bvecs

### Data preprocessing

The diffusion data after normalization (alignment and orientation) are stored as follows:

brain-life.app-dtiinit/sub-{}/dwi/

sub-{}_tag-normalized_tag-singleshell_tag-dtiinit_desc-{}_dwi.json

sub-{}_tag-normalized_tag-singleshell_tag-dtiinit_desc-{}_dwi.nii.gz

sub-{}_tag-normalized_tag-singleshell_tag-dtiinit_desc-{}_dwi.bvals

sub-{}_tag-normalized_tag-singleshell_tag-dtiinit_desc-{}_dwi.bvecs

### Tractography

The diffusivity signal reconstruction models generated the following volumetric images as NifTI files: fractional anisotropy (_FA.nii.gz), the diffusion tensor model (model-DTI) and the constrained spherical deconvolution model (model-CSD). A brain mask and a white matter mask are also distributed at the dMRI data resolution (type-Brain, type-Whitematter). To increase impact and compatibility of the O3D data files, two copies of each tractogram are distributed, one in MRtrix format (tck) and the other TrackVis format (trk). One file is outputted per repeated-measure tractogram, and tractography method (tag-dtstream, tag-sdstream, tag-sddprob).

brain-life.app-tracking/sub-{}/dwi/

sub-{}_run-{}_desc-{}_FA.nii.gz

sub-{}_run-{}_desc-{}_model-DTI_diffmodel.nii.gz

sub-{}_run-{}_desc-{}_model-CSD_diffmodel.nii.gz

sub-{}_run-{}_desc-{}_type-Brain_mask.nii.gz

sub-{}_run-{}_desc-{}_type-Whitematter_mask.nii.gz

sub-{}_run-{}_tag-dtstream_desc-{}_tractography.json

sub-{}_run-{}_tag-dtstream_desc-{}_tractography.tck

sub-{}_run-{}_tag-sdstream_desc-{}_tractography.json

sub-{}_run-{}_tag-sdstream_desc-{}_tractography.tck

sub-{}_run-{}_tag-sdprob_desc-{}_tractography.json

sub-{}_run-{}_tag-sdprob_desc-{}_tractography.tck

brainlife.app-convert-tck-to-trk/sub-{}/dwi/

sub-{}_run-{}_tag-dtstream_tag-dwi_desc-{}_tractography.json

sub-{}_run-{}_tag-dtstream_tag-dwi_desc-{}_tractography.trk

sub-{}_run-{}_tag-sdstream_tag-dwi_desc-{}_tractography.json

sub-{}_run-{}_tag-sdstream_tag-dwi_desc-{}_tractography.trk

sub-{}_run-{}_tag-sdprob_tag-dwi_desc-{}_tractography.json

sub-{}_run-{}_tag-sdprob_tag-dwi_desc-{}_tractography.trk

### Tractography evaluation

We used the Linear Fascicle Model to evaluate the quality of fit of the dMRI signal. The output of LiFE tractography evaluation process is stored as an *encode* model structure^22^. The encode brainlife.io DataType is stored as matlab structure (_life.mat). A detailed documentation of encode model structure is available at github.com/brain-life/encode. One encode model structure per repeated-measure tractogram is distributed, for a total of ten runs, one for each tractography algorithm.

brain-life.app-life/sub-{}/dwi/

sub-{}_run-{}_tag-dtstream_desc-{}_life.json

sub-{}_run-{}_tag-dtstream_desc-{}_life.mat

sub-{}_run-{}_tag-sdstream_desc-{}_life.json

sub-{}_run-{}_tag-sdstream_desc-{}_life.mat

sub-{}_run-{}_tag-sdprob_desc-{}_life.json

sub-{}_run-{}_tag-sdprob_desc-{}_life.mat

### White matter classification

Twenty human major white matter tracts were classified for each Tractogram and are distributed using the TRK file format. A json file for each tractogram records for each tract the enumeration ID, the label of the tract and the number of fibers.

brainlife.app-wmctotrk/sub-{}/dwi/

sub-{}_run-{}_tag-dtstream_tag-afq_tag-cleaned_tag_wmc_\

desc-run-{}_tractography.trk

sub-{}_run-{}_tag-dtstream_tag-afq_tag-cleaned_tag_wmc_\

desc-run-{}_tractography.json

sub-{}_run-{}_tag-sdstream_tag-afq_tag-cleaned_tag_wmc_\

desc-run-{}_tractography.trk

sub-{}_run-{}_tag-sdstream_tag-afq_tag-cleaned_tag_wmc_\

desc-run-{}_tractography.json

sub-{}_run-{}_tag-sdprob_tag-afq_tag-cleaned_tag_wmc_\

desc-run-{}_tractography.trk

sub-{}_run-{}_tag-sdprob_tag-afq_tag-cleaned_tag_wmc_\

desc-run-{}_tractography.json

### Connectome matrices

Connection matrices were built using the aforementioned tractograms and the Desikan-Killiany Atlas from FreeSurfer^91^. A connection matrix was computed for each repeated-measure tractogram, processed using the LiFE method, and for each tractography method {dtstream, sdstream, sddprob}. Two measures of connectivity were computed: fiber count and fiber density (fiber count divided by the volume of the two termination areas^119,123^). Connection matrices are stored as pairs of.csv and.json files. A NifTI file records the cortical parcellation used to define the ROIs of the networks.

brain-life.app-networkneuro/sub-{}/dwi/

sub-{}_run-{}_tag-{}_desc-{}_connectivity.json

sub-{}_run-{}_tag-{}_desc-{}_tag-count_connectivity.csv

sub-{}_run-{}_tag-{}_desc-{}_tag-density_connectivity.csv

sub-{}_run-{}_tag-{}_desc-{}_label-GM_dseg.nii.gz

## Technical Validation

In this section we provide both a qualitative and quantitative evaluation of the data derivatives made available at^43^. We show data SNR in each dataset used, demonstrate quality of alignment between dMRI and anatomy files, and show the diffusion signal in the voxel reconstruction, several properties of the tractography models and of the major white matter tracts segmented.

### Data preprocessing

**Fig. 1:**
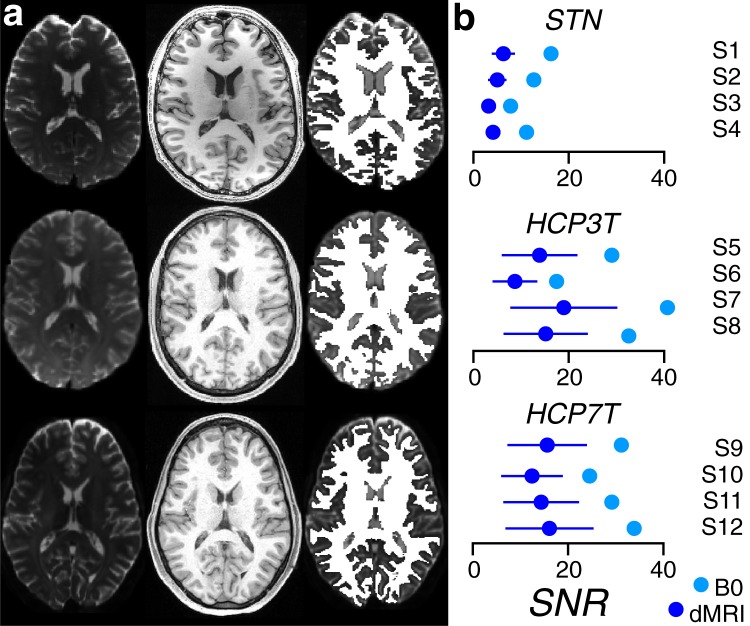
Data quality and preprocessing. (**a**) Axial view of dMRI (left, non-diffusion weighted volume, B0), aligned anatomical image (center) and white matter mask obtained from the anatomy (white), overlaid on the B0 to show the quality of the white matter volume delineation. One example subject is reproduced from the Stanford (top), Human Connectome 3T (middle) and Human Connectome 7T (bottom) data. (**b**) Mean and ±1 sd across diffusion-weighted measurements of the signal-to-noise (SNR) for each subject and dataset in the O3D distribution as implemented at^126^.

Data preprocessing was performed using a combination of previously published pipelines^22,40,45–47^ (see Methods for additional details). Diffusion weighted MRI data were aligned to the T1-weighted anatomical images (Fig. 1a left-hand columns, see Methods for additional details). The T1w images were used to segment the brain into different tissue types and brain regions^72^. The total white matter volume was identified using the previously generated white matter tissue segmentation and all subsequent analyses were performed within the white matter volume. Figure 1a shows how the white matter volume (mask) defined on the anatomical image (middle) aligns with the non diffusion-weighted signal (B_0_) image of the diffusion MRI data (left-hand panel) in three example subjects one per dataset.

To compare dMRI data quality across datasets we computed the signal-to-noise ratio (SNR) comparing the mean attenuated dMRi signal to the background noise for both diffusion-weighted and B_0_ measurements (Fig. 1b), as described by^124,125^. The brainlife.io App implementing this SNR method can be found at^126^.

### White matter microstructure reconstruction within the voxel

**Fig. 2:**
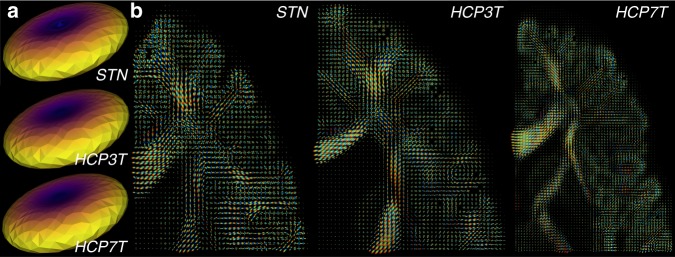
Estimated fiber orientation distribution functions (fODF). (**a**) Examples of estimated single-fiber response function used to compute the fODF individually in each subject. The similarity and flat shape of the response functions ensures model-fit quality^110,111^. (**b**) Axial brain views from three example subjects in each dataset depicting the estimated fODF (fiber orientation distribution functions) in a series of voxels covering the corpus callosum and the central-semiovale. Coverage of the response functions and orientation are consistent with major anatomical understanding.

The dMRI signal within each voxel was reconstructed using the two dominant models, namely the diffusion tensor (DTI^127^) and constrained-spherical deconvolution (CSD^110,111^). Specifically, when applying CSD, we utilized an *L*_max_ parameter of 10 for STN and 8 for HCP. These models provide different opportunities as well as limitations to characterize the dMRI signal and brain fibers. Figure 2 shows the quality of the estimated deconvolution kernel (a) and the fit of the CSD model in three representative axial brain slices, one per dataset (b). The kernel estimation is important for effective fiber distribution estimation and long-range tracking^128^. Both dMRI reconstructions (DTI and CSD) have been manually curated by visual inspection to assure quality in the O3D dataset.

### Tractography

Tractography was reconstructed using two established methods: deterministic and probabilistic^76,106–108,129–131^ tractography. We used Deterministic tractography either in combination with DTI or CSD models. Probabilistic tractography was only used in combination with the CSD model. It has been established that application of these different methods result in the generation of white matter fascicles with different anatomical properties^29,40,47,54,132–134^. The O3D dataset provides three tractography reconstructions for each individual brain. Tractography outputs were stored using common file formats (.tck and.trk) to allow investigators to compare, reuse and improve upon current tracking methods.

**Fig. 3:**
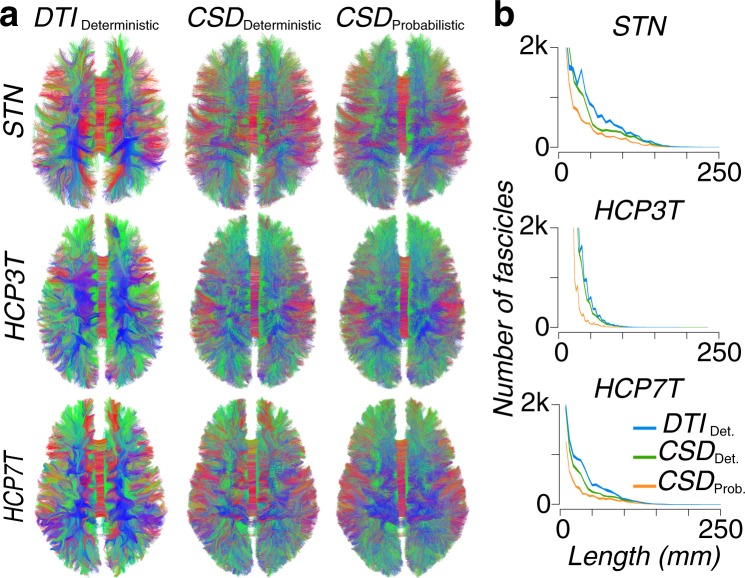
Visualization of whole-brain tractograms and fascicle length distribution. (**a**) The full brain tractography for each of the three datasets, as generated using *DTI*_deterministic_, *CSD*_deterministic_ and *CSD*_probabilistic_ Models. (**b**) The whole-brain connectome streamline count for each of the three tractography models applied to the STN, HCP3T and HCP7T datasets.

Figure 3a provides a qualitative depiction of the whole-brain tractography reconstruction in a subject from each dataset. Figure 3b reports a quantitative comparison of the fascicles length distribution for whole brain tractograms in the three example subjects in Fig. 3a.

### Human major white matter tracts

**Fig. 4:**
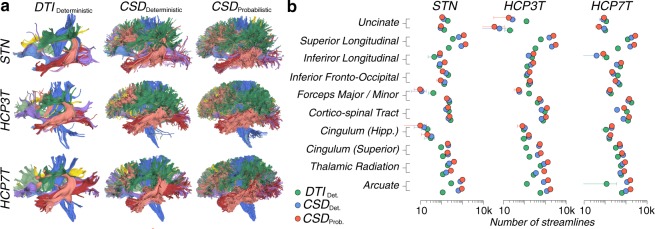
Anatomy of tracts and number of fascicles per tract. (**a**) The morphologies of several major tracts, overlaid with one another, as segmented from whole brain connectomes. Tractography generated for each dataset using *DTI*_deterministic_, *CSD*_deterministic_ and *CSD*_probabilistic_ models. Colors correspond to individual tracts. (**b**) The streamline counts associated with several major tracts. Marker color corresponds to tractography model. Error bars generated from standard deviation across ten replications.

We report a qualitative visualization of the eleven major white matter tracts which were segmented from each connectome. These correspond to nine major tracts in the left and right hemispheres and two cross-hemispheric tracts. These tracts were segmented using a standardized methodology and atlases^75,90,135^. Files are saved as.tck and.trk file formats. Previous work has shown that the application of different tractography models results in anatomical tracts with different morphologies, volumes and streamline counts^22,29,40,47,54^. Figure 4a depicts these tracts as segmented for each subject, using each diffusion model, with colors corresponding to specific tracts. Figure 4b plots the number of streamlines, from the source whole brain tractogram, identified as constituting each of these major tracts.

### Network neuroscience

**Fig. 5:**
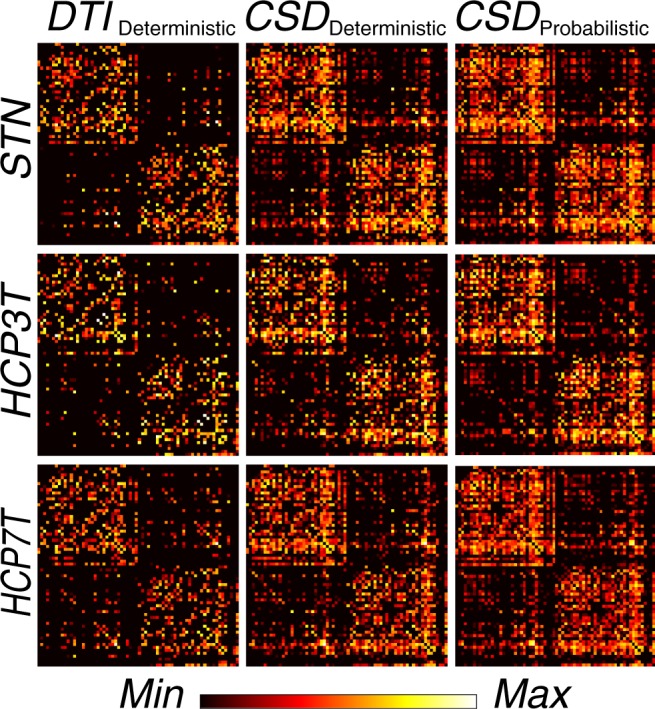
Brain network matrices. Nine representative matrices of connectivity between anatomical regions defined in the Desikan-Killiany atlas^91^. Matrices report fiber density computed as twice the number of streamlines touching a pair of regions divided by the combined size of the two regions (in number of brain voxels). Density is normalized across matrices, brighter colors indicate higher density. Networks depicted were generated for three representative subjects, one per dataset, using *DTI*_deterministic_, *CSD*_deterministic_ and *CSD*_probabilistic_ tractography.

The aforementioned whole brain tractograms represent a model of how the white matter of the brain connects cortical regions to one another. Together with a cortical parcellation, this rich body of connectivity information can be summarized into a network matrix, with brain regions or regions of interest representing network nodes, and measures related to connection weight or density corresponding to network edges. Graphical summaries like those presented in Fig. 5 provide a common way to visualize these connectivity patterns. This graph or network representation of connectomes enables a large array of analytic and modeling tools to probe connectivity motifs, modularity, centrality, vulnerability and other network or graph-theoretic measures^63,136–138^. The O3D dataset features structural connectivity data, arranged as matrices, along with the numeric key indicating the cortical parcels names for each network node. Connectivity matrices were computed using two edge metrics: streamline count and streamline density^88,119,123^.

## Usage Notes

The O3D dataset is publicly available at the link provided in^43^. Data files can be downloaded organized according to the BIDS^85^ standard. Different data derivatives are distributed with formats, such as NifTI, TCK, TRK or plain text. Access to the published data is currently supported via (i) web interface and (ii) Command Line Interface (CLI).

The brainlife.io CLI can be installed on most Unix/Linux systems using the following command: npm install brainlife -g. The CLI can be used to query and download partial of full datasets. The following example shows the CLI command to download all T1w datasets from a subject in the publication data Release 2:

bl pub query # this will return the publication IDs

bl bids download --pub 5c0ff604391ed50032b634d1 --subject 0001 --datatype neuro/anat/t1w

The following command downloads the data in the entire project (from Release 2) into BIDS format:

bl bids download --pub 5c0ff604391ed50032b634d1

Additional information about the brainlife.io CLI commands can be found at https://github.com/brainlife/cli In addition, https://brainlife.io/project/5a022fc99c0d250055709e9c/detail is the project page with read-only data supporting browsing, visualization, download or additional processing. O3D uses the data originated from projects with different license and user terms. The four datasets (subject 1–4) originated from the Stanford University project are distributed with CC-BY license (creativecommons.org/licenses/by/4.0/). Access to the eight datasets originated from the Human Connectome Project (subject 5–12) require that users agree to the HCP Data Use Terms humanconnectome.org/study/hcp-young-adult/data-use-terms.

## ISA-Tab metadata file

Download metadata file
